# Telomere biology is differently affected within clinical subsets of systemic sclerosis and points towards different downstream defects

**DOI:** 10.1186/1479-5876-10-S3-P37

**Published:** 2012-11-28

**Authors:** Jasper CA Broen, Liane McGlynn, Meng May Chee, Lenny Geurts-van Bon, Robert L Lafyatis, Timothy RDJ Radstake, Paul G Shiels

**Affiliations:** 1 Dept. of Rheumatology and Clinical Immunology, University Utrecht Medical Center, Utrecht, the Netherlands; 2 Dept. of Cancer Sciences, University of Glasgow, Glasgow, Scotland, UK; 3 Dept. of Rheumatology, Boston University Medical Center, Boston, MA, USA

## Background

Systemic sclerosis is a immune mediated inflammatory disease culminating in vasculopathy and extensive fibrosis of the skin and internal organs. Telomere shortening has previously been described in SSc.

## Aim

To replicate previous findings in large cohort, investigate telomere shortening in multiple immune celltypes and scrutinize underlying aberrances in telosome gene expression.

## Methods

We measured telomere length by PCR in a cohort of 185 SSc patients and 100 healthy controls. Next we investigated plasmacytoid dendritic cells, T cells, B cells, monocytes and myeloid dendritic cells from 25 SSc patients for cell specific telomere attrition. Finally we investigated whether there were differences in expression of 31 genes involved in telomere pathways.

## Results

We observed a significant age related telomere attrition in healthy controls and lcSSc patients (Both *p*< 0.001), but not in dcSSc patients. In the immune cell subset specific analysis we observed significant shorter telomeres in B cells and myeloid dendritic cells of both lcSSc and dcSSc patients (B-Cells *p*=*0.014*, *p*=*0.002* & myDCs *p*=*0.019*, *p*=*0.004* respectively). PDCs and T cells were significantly shorter in dcSSc patients only (p=0.001 and p=0.003 respectively). In addition, we observed in early SSc, that B cells exhibit a significant upregulation of the telosome genes SIRT6, RIF1 and WRN (after correction for multiple testing p=0.03, 0.006 and 0.048 respectively). In later disease there is a significant higher expression of HDAC9 in monocytes from dcSSc compared to lcSSc patients. Intriguingly, in PDCs of diffuse SSc patients, regardless whether it is early or progressed disease the expression of SIRT1 is significantly lower (p=0.002 in all comparisons).

## Conclusions

Aberrances in telomere shortening and biology are a feature of SSc, reflecting a difference in clinical subsets at the cellular level.

